# Protective Effects of Clenbuterol against Dexamethasone-Induced Masseter Muscle Atrophy and Myosin Heavy Chain Transition

**DOI:** 10.1371/journal.pone.0128263

**Published:** 2015-06-08

**Authors:** Daisuke Umeki, Yoshiki Ohnuki, Yasumasa Mototani, Kouichi Shiozawa, Kenji Suita, Takayuki Fujita, Yoshiki Nakamura, Yasutake Saeki, Satoshi Okumura

**Affiliations:** 1 Department of Physiology, Tsurumi University School of Dental Medicine, 230–8501, Yokohama, Japan; 2 Department of Orthodontics, Tsurumi University School of Dental Medicine, 230–8501, Yokohama, Japan; 3 Cardiovascular Research Institute, Yokohama City University Graduate School of Medicine, Yokohama, 236–0004, Japan; University of Louisville School of Medicine, UNITED STATES

## Abstract

**Background:**

Glucocorticoid has a direct catabolic effect on skeletal muscle, leading to muscle atrophy, but no effective pharmacotherapy is available. We reported that clenbuterol (CB) induced masseter muscle hypertrophy and slow-to-fast myosin heavy chain (MHC) isoform transition through direct muscle β2-adrenergic receptor stimulation. Thus, we hypothesized that CB would antagonize glucocorticoid (dexamethasone; DEX)-induced muscle atrophy and fast-to-slow MHC isoform transition.

**Methodology:**

We examined the effect of CB on DEX-induced masseter muscle atrophy by measuring masseter muscle weight, fiber diameter, cross-sectional area, and myosin heavy chain (MHC) composition. To elucidate the mechanisms involved, we used immunoblotting to study the effects of CB on muscle hypertrophic signaling (insulin growth factor 1 (IGF1) expression, Akt/mammalian target of rapamycin (mTOR) pathway, and calcineurin pathway) and atrophic signaling (Akt/Forkhead box-O (FOXO) pathway and myostatin expression) in masseter muscle of rats treated with DEX and/or CB.

**Results and Conclusion:**

Masseter muscle weight in the DEX-treated group was significantly lower than that in the Control group, as expected, but co-treatment with CB suppressed the DEX-induced masseter muscle atrophy, concomitantly with inhibition of fast-to-slow MHC isoforms transition. Activation of the Akt/mTOR pathway in masseter muscle of the DEX-treated group was significantly inhibited compared to that of the Control group, and CB suppressed this inhibition. DEX also suppressed expression of IGF1 (positive regulator of muscle growth), and CB attenuated this inhibition. Myostatin protein expression was unchanged. CB had no effect on activation of the Akt/FOXO pathway. These results indicate that CB antagonizes DEX-induced muscle atrophy and fast-to-slow MHC isoform transition via modulation of Akt/mTOR activity and IGF1 expression. CB might be a useful pharmacological agent for treatment of glucocorticoid-induced muscle atrophy.

## Introduction

β_2_-agonists and glucocorticoids exert opposite effects, i.e., β_2_-agonists promote skeletal muscle hypertrophy and are used as anabolic drugs to increase skeletal muscle weight, whereas glucocorticoids induce myopathy, characterized by muscle weakness, atrophy, and fatigue [1,2]. Furthermore, β_2_-agonists induce slow-to-fast myosin heavy chain (MHC) isoform transition [3–5], while glucocorticoids induce fast-to-slow MHC isoform transition [6]. Glucocorticoids, such as dexamethasone (DEX) are potent immunosuppressants and anti-inflammatory agents, and are widely used to treat various clinical conditions, including asthma and autoimmune diseases. However, glucocorticoid-induced myopathy is a serious side effect, and indeed is the most common type of drug-induced myopathy. At present, no pharmacological treatment, other than dosage reduction or withdrawal of glucocorticoid, is available for glucocorticoid-induced myopathy. Therefore, it is important to understand the interaction of β_2_-agonists and glucocorticoids in skeletal muscle in order to provide potential treatment options for glucocorticoid-induced myopathy.

Several previous studies have examined the effects of the β_2_-agonist clenbuterol (CB) on DEX-induced muscle atrophy. In mice, CB (4mg/kg) partially blocked DEX (5mg/kg)-induced muscle atrophy of soleus, gastrocnemius and extensor digitorum longus muscle, and atrophy was completely prevented by increasing the concentration to 8mg/kg [4]. In rats, CB (2mg/kg for 2 weeks) was reported to minimize diaphragm atrophy induced by DEX (3mg/kg for 2 weeks), although it did not show a protective effect against DEX-induced diaphragm dysfunction [7]. It was also reported that CB (4mg/kg for 10 days) partially inhibited DEX (2mg/kg for 10 days)-induced atrophy of hind-limb muscle in rats [8]. Taken together, these results suggest that CB might be protective against DEX-induced muscle atrophy, however nothing is known about the mechanisms of these putative effects. Therefore, in order to clarify the mechanism(s) involved, we examined the changes of hypertrophic signaling (insulin growth factor 1 (IGF1) expression, Akt/mTOR pathway, and calcineurin pathway) and atrophic signaling (Akt/Forkhead box-O (FOXO) pathway and myostatin expression) in masseter muscle of rats treated with DEX and/or CB.

## Materials and Methods

### Animals

Animals were treated in accordance with institutional guidelines, and the experimental protocol was approved by the Animal Care and Use Committee of Tsurumi University. Wister rats, aged 8 weeks, were given a regular diet (CE-2: 344.9 kcal/100g; CLEA Japan, Inc. Tokyo, Japan), and were divided into four groups: a normal control group (Control), a CB (Sigma, St. Louis, MO, USA)-treated group (CB), a DEX-treated group (Sigma, St. Louis, MO, USA) (DEX), and a DEX plus CB-treated group (CB+DEX). CB was directly dissolved in drinking water (30 mg/L; freshly prepared every day) and administered for 2 weeks. DEX was administered intraperitoneally (i.p.) for 2 weeks (6mg/kg/every other day), concomitantly with the CB treatment [6]. Body weight, food and water intake were monitored for all animals throughout the 2-week experimental period.

Oral administration of CB at this dose (30 mg/L) increased masseter muscle volume time-dependently, with a maximum at two weeks after administration. Several reports indicate that 30mg/L is the most effective dose for promoting maximal growth and two weeks is a suitable time for evaluation, and thus we adopted this dosing protocol for examining the effects of DEX on CB-mediated skeletal muscle hypertrophy [9–12].

Numbers of animals used in different analyses ranged from 3 to 11, in large part because the data were derived from two series of experiments. The total number of animals in the first series of experiments was 44 (n = 11/group) and that in the second series of experiments, conducted subsequently to clarify various issues, was 20 (n = 5/per group). After completion of each treatment, all animals were weighted and killed by exsanguination under anesthesia with a fatal overdose of pentobarbital sodium (50 mg/kg/body weight) [13]. The left and right masseter, tibialis anterior, and soleus muscles, as well as heart muscle, were excised and weighted. The central portion of the left superficial masseter muscle was divided into two pieces, which were rapidly frozen in liquid nitrogen and stored at -85°C.

Analyses of body weight (n = 6–9/group), daily consumption of food and water (n = 9/group each), daily intake of CB and energy (n = 9/group each), masseter muscle mass (n = 6–9/group), SDS-PAGE (n = 5–8/group) and real-time quantitative PCR (n = 8–10/group) were performed using samples obtained in the first series of experiments. Subsequent western blotting analysis (n = 3–5/group) and measurements of the mass of tibialis anterior, soleus, and heart muscles (n = 3/group each) were performed using samples obtained in the second series of experiments.

The central portion of the right superficial masseter muscle was fixed in 4% (w/v) paraformaldehyde in phosphate-buffered saline (PBS) for 2 hours at 4°C, and the fixed specimens were washed with PBS and then stored in 40% (w/v) sucrose in PBS at 4°C for hematoxylin and eosin (HE) staining. Analyses of fiber diameter (n = 11/group each) and cross sectional area (CSA) (n = 4/group each) were performed using samples prepared in the first series of experiments.

### Histological analysis

Cross sections (10 μm thick) were cut from the middle portion of the right masseter muscle with a cryostat (CM1900, Leica Microsystems, Nussloch, Germany) at -20°C. The sections were stained with hematoxylin and eosin (HE) and observed under a light microscope (BX61, Olympus Co., Tokyo, Japan) [14]. Micrographs were taken with a digital camera (DP-72, Olympus Co.) connected to a personal computer. The cross-sectional size of muscle fibers was evaluated by measuring the minimal diameter [15] and the CSA [16] of muscle fibers with image analysis software (Image J 1.45) and averaged to obtain the mean value in each rat.

### MHC composition

MHC composition was analyzed by means of SDS-polyacrylamide gel electrophoresis (SDS-PAGE) in the left superficial masseter muscles excised from the four groups, as described previously [15,17].

### Western blotting

Masseter muscle excised from the rats that had been treated with CB and/or DEX for 7–14 days, was homogenized in a Polytron (Kinematica AG, Lucerne, Switzerland) in ice-cold RIPA buffer (Thermo Fisher Scientific, Waltham, MA, USA: 25 mM Tris-HCl (pH 7.6), 150 mM NaCl, 1% NP-40, 1% sodium deoxycholate, 0.1% SDS) without addition of inhibitors [18], and the homogenate was centrifuged at 13,000 x *g* for 10 min at 4°C. The supernatant was collected and the protein concentration was measured using a DC protein assay kit (Bio-Rad, Hercules, CA, USA). Equal amounts of protein (5 μg) were subjected to 12.5% SDS-PAGE and blotted onto 0.2 mm PVDF membrane (Millipore, Billerica, MA, USA).

Western blotting was conducted with commercially available antibodies [19–23]. The primary antibodies against glucocorticoid receptor (#12041), Akt (#9272), phospho-Akt (Ser-473, #9271), 70-kDa ribosomal S6 kinase 1 (S6K1) (#9202), phospho-S6K1 (Thr-389, #9205), FOXO1 (#2880), phospho-FOXO1 (Ser-256, #9461), FOXO3a (#12829), phospho-FOXO3a (Ser-253, #13129) were purchased from Cell Signaling Technology (Boston, MA, USA) and the primary antibodies against β_2_-adrenergic receptor (β_2_-AR) (sc-569), insulin growth factor 1 (IGF1) (sc-9013), myostatin (sc-6885-R), glyceraldehyde 3-phosphate dehydrogenase (GAPDH) (sc-25778), nuclear factor of activated T cells (NFAT) c1 (sc-13033), phospho-NFATc1 (Ser-259, sc-32979), NFATc3 (sc-8321), phospho-NFATc3 (Ser-265, sc-32982), and modulatory calcineurin-interacting protein 1 (sc-66864) were purchased from Santa Cruz Biotechnology (Santa Cruz, CA, USA). Horseradish peroxidase-conjugated anti-rabbit IgG (GB Healthcare, NA934) was used as a secondary antibody. The primary and secondary antibodies were diluted in Tris-buffered saline (pH 7.6) with 0.1% Tween 20 and 5% bovine serum albumin. The blots were visualized with enhanced chemiluminescence solution (ECL Prime Western Blotting Detection Reagent, GE Healthware, Piscataway, NJ, USA) and scanned with a densitometer (LAS-1000, Fuji Photo Film, Tokyo, Japan).

### Real-time quantitative PCR

Total RNA was isolated from individual samples taken from left superficial masseter muscle in each group according to the manufacturer’s instructions (FastRNATM Kit-GREEN; BIO 101, Vista, CA, USA). Real-time quantitative PCR for atrogin-1 and muscle Ring Finger-1 (MuRF1) was performed with oligonucleotide primer sets based on published sequences.

Atrogin-1 (product size: 196 bp) [24]

(sense) 5’-GATGAGAAAAGCGGCACCTTCGT-3’

(antisense) 5’-ATCCATGGCGCTCCTTAGTACTCCC-3’

MuRF1 (product size: 130 bp) [25]

(sense) 5’-AGGACAACCTCGTGCCTACAAG-3’

(antisense) 5’-ACAACCTGTGCCGCAAGTG-3’

S16 (product size: 178 bp) [26]

(sense) 5’-CGTGCAGGTCTTCGGACGCA-3’

(antisense) 5’-CCGAATATCCACACCAGCAA-3’

### Statistical analysis

**Fig 1:**
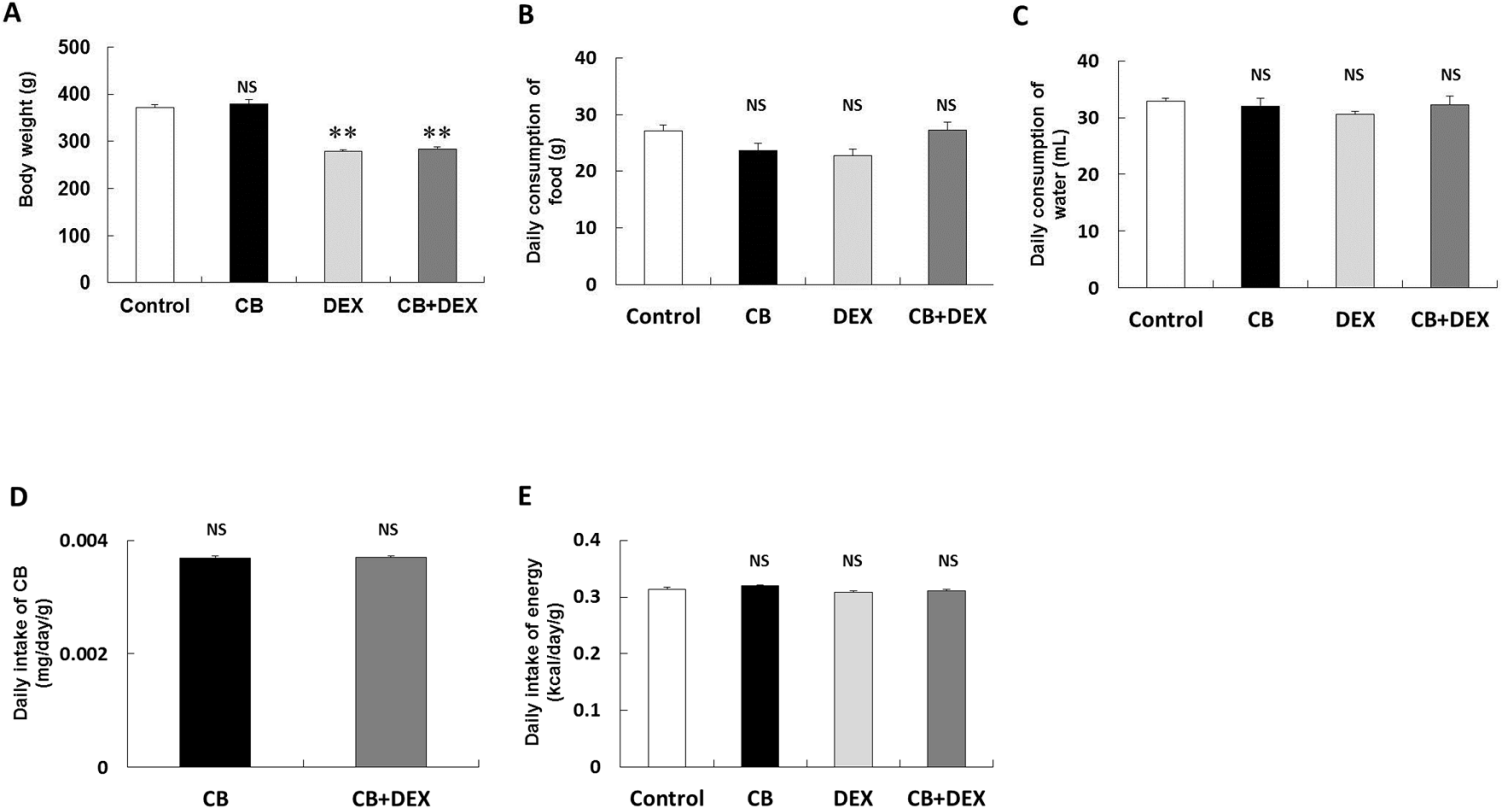
Changes of body weight, daily consumption of food and water, and daily intake of CB and energy. (**A)** Body weight (BW: g) of CB was similar to the Control (*P* = NS vs. Control). On the other hand, BW of both the DEX and CB+DEX groups was significantly smaller than that in the Control (**P < 0.01 vs. Control in each case). **(B-C)** No significant difference in daily consumption of food **(B)** or water **(C)** was observed among the CB, DEX, CB+DEX, and the Control groups (*P* = NS vs. Control in each case).**(D-E)** No significant difference was observed in daily intake of CB per baseline BW between the CB and CB+DEX groups **(D)** or in energy intake among the CB, DEX, CB+DEX, and the Control groups **(E)** (*P* = NS vs. Control in each case). The values of BW, consumption of food, consumption of water, CB intake, and energy intake in the Control group were taken as 100% in each determination.

**Fig 2:**
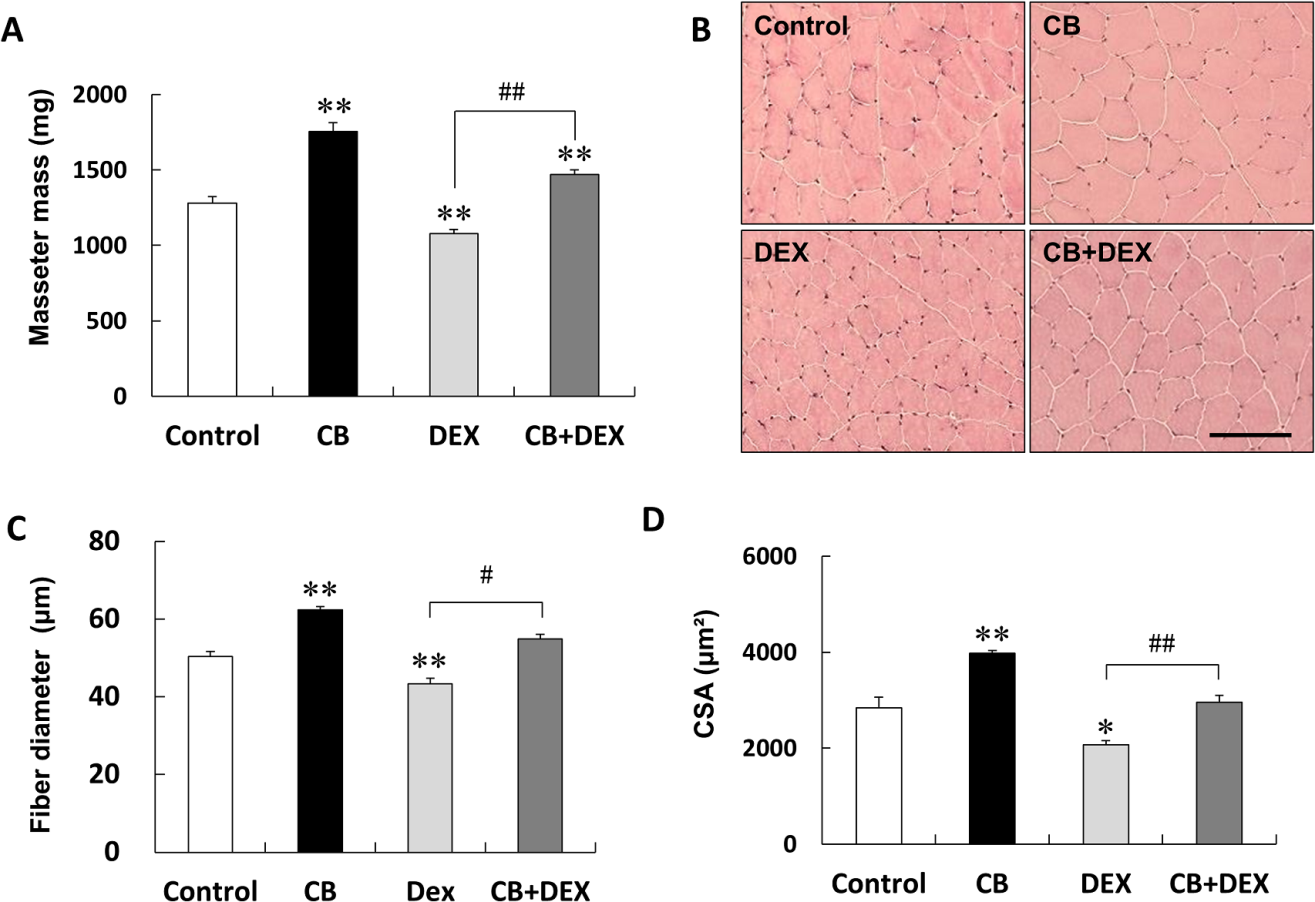
Masseter muscle hypertrophy after treatment with CB and/or DEX for 2 weeks. **(A)** Masseter muscle mass weight of CB was significantly greater, and that of DEX was significantly smaller than that of the Control (**P < 0.01 vs. Control in each case). Importantly, the DEX-mediated inhibition was suppressed by co-treatment with CB (CB+DEX vs. DEX, ^##^ *P* < 0.01). **(B)** Representative images of HE staining of masseter muscle of rats in the Control, CB, DEX, and CB+DEX groups. *Scale bar*: 100 μm. **(C)** Fiber diameter of CB was significantly greater and that of DEX was significantly smaller than that of the Control (**P < 0.01 vs. Control in each case). DEX-mediated decrease was suppressed by co-treatment with CB (CB+DEX vs. DEX, ^#^ *P* < 0.05). **(D)** CSA of CB was significantly greater (**P < 0.01) and that of DEX was significantly smaller (*P < 0.05) than that of the Control. DEX-mediated inhibition was suppressed by the co-treatment of CB (^##^ *P* < 0.01 vs. DEX). Masseter mass, fiber diameter and CSA in the Control were taken as 100% in each determination.

**Fig 3:**
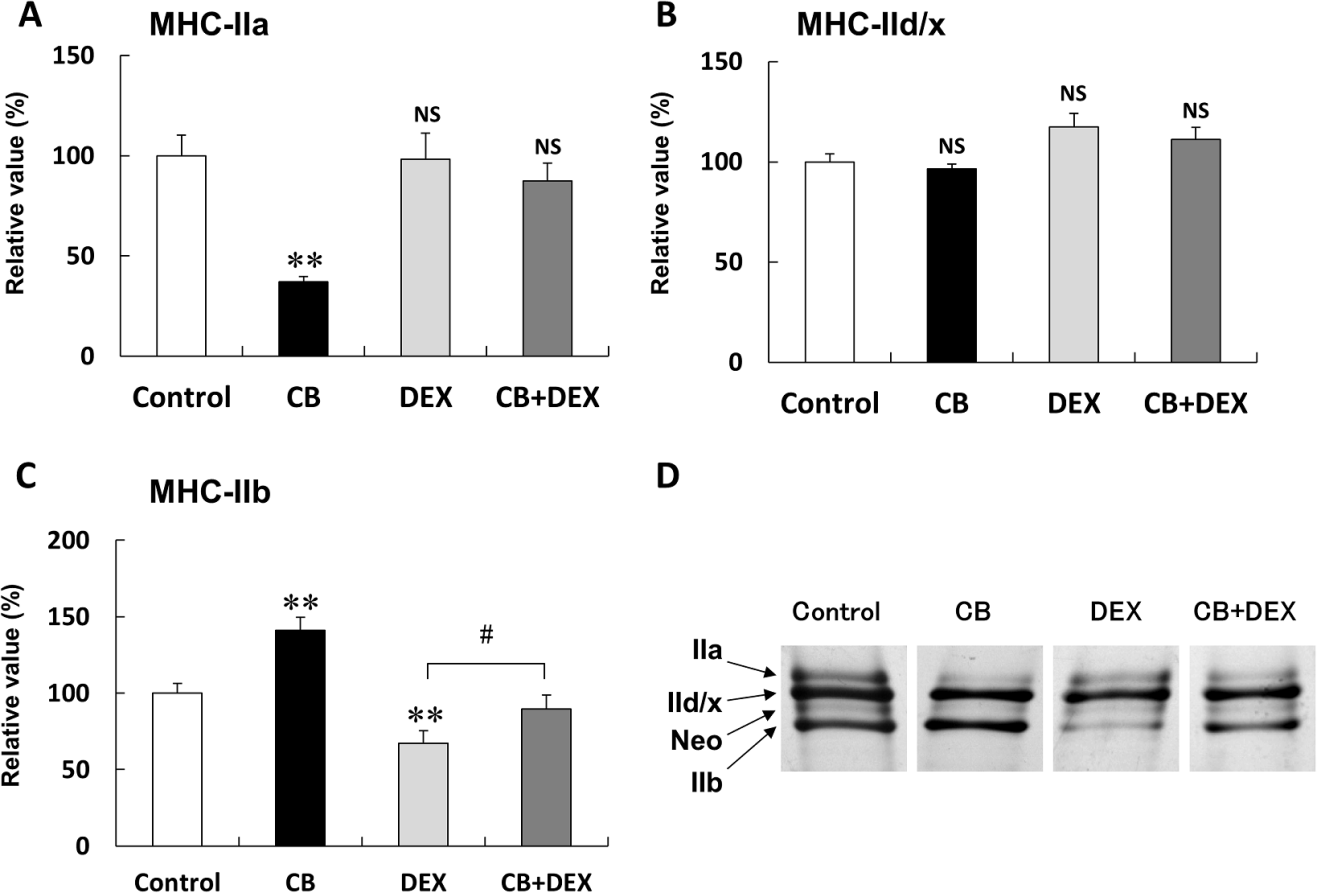
Changes in composition of MHC isoforms in masseter muscle after treatment with CB and/or DEX for 2 weeks. **(A)** Protein expression level of MHC-IIa in the CB group was significantly smaller than that of the Control (**P < 0.01), while those in the DEX and CB+DEX groups were similar to the Control (*P* = NS vs. Control in each case) **(B)** Protein expression level of MHC-IId/x in the CB group was similar to that of the Control (*P* = NS), while those of MHC-IId/x in the DEX and CB+DEX groups tended to be greater than that of the Control, though without significance (*P* = NS vs. Control in each case). **(C)** Protein expression level of MHC-IIb in the CB group was significantly greater and that in the DEX group was significantly smaller than that in the Control group (**P < 0.01 vs. Control in each case). The DEX-mediated decrease of MHC-IIb was suppressed by co-treatment with CB (^#^ *P* < 0.05). **(D)** Representative silver staining of MHC isoforms in the masseter muscle. Expression of MHC-I was not observed. The density of the MHC-Neo band was too low to permit quantification. The average amount of each MHC isoform expression in the Control was taken as 100% in each determination.

**Fig 4:**
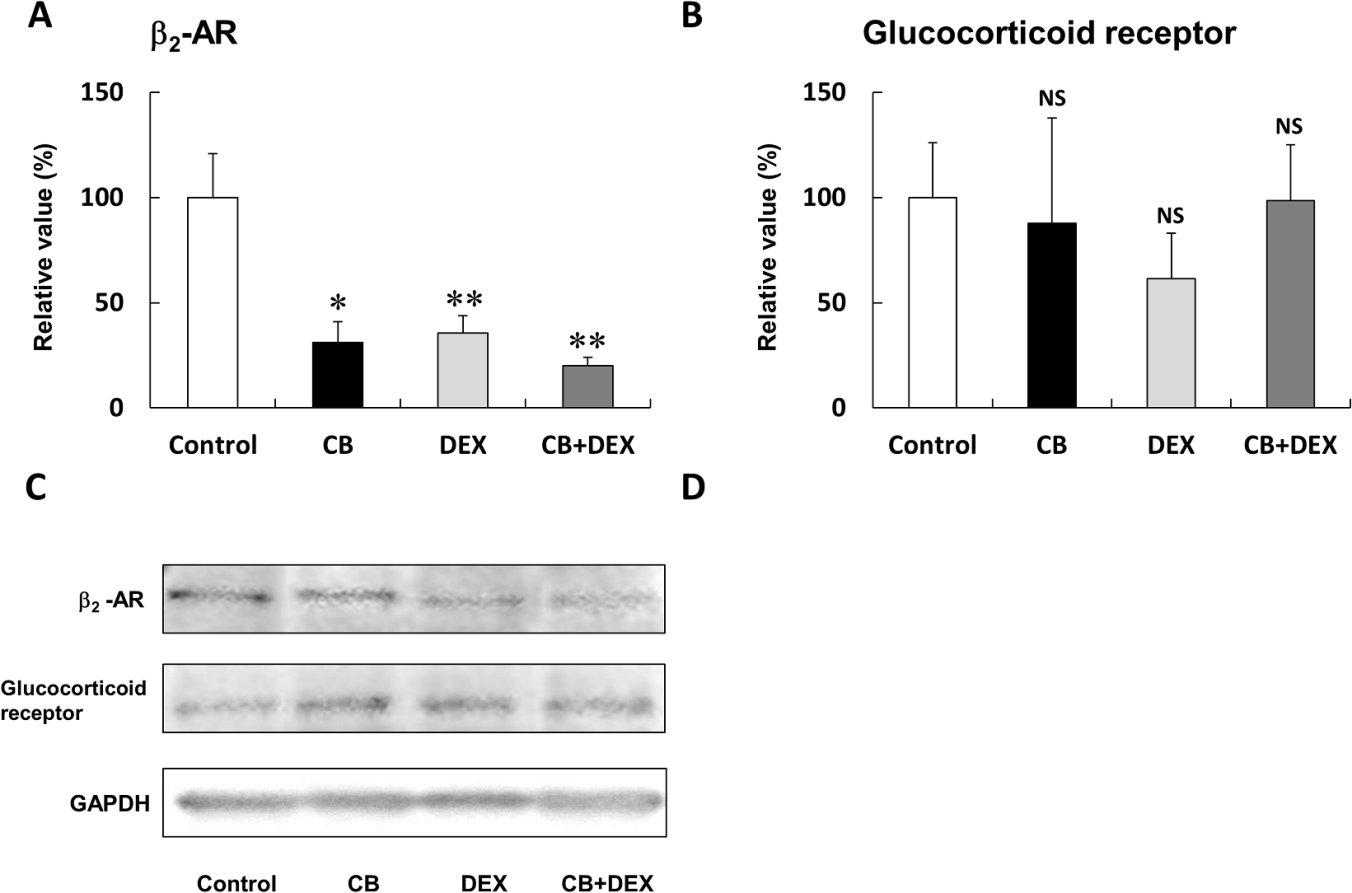
Changes in expression of β_2_-AR and glucocorticoid receptor after treatment with CB and/or DEX for 2 weeks. **(A)** Expression of β_**2**_-AR protein in masseter muscle of the CB, DEX, and CB+DEX groups was smaller than that in the Control (*P < 0.05, **P < 0.01 vs. Control), but the magnitude of the attenuation was similar among the three groups. **(B)** There was no change in expression of glucocorticoid receptor protein in the CB, DEX, and CB+DEX groups (*P* = NS vs. Control in each case). **(C)** Representative immunoblotting results for β_**2**_-AR and glucocorticoid receptor. The amount of expression in the Control was taken as 100% in each determination. GAPDH; glyceraldehyde 3-phosphate dehydrogenase

**Fig 5:**
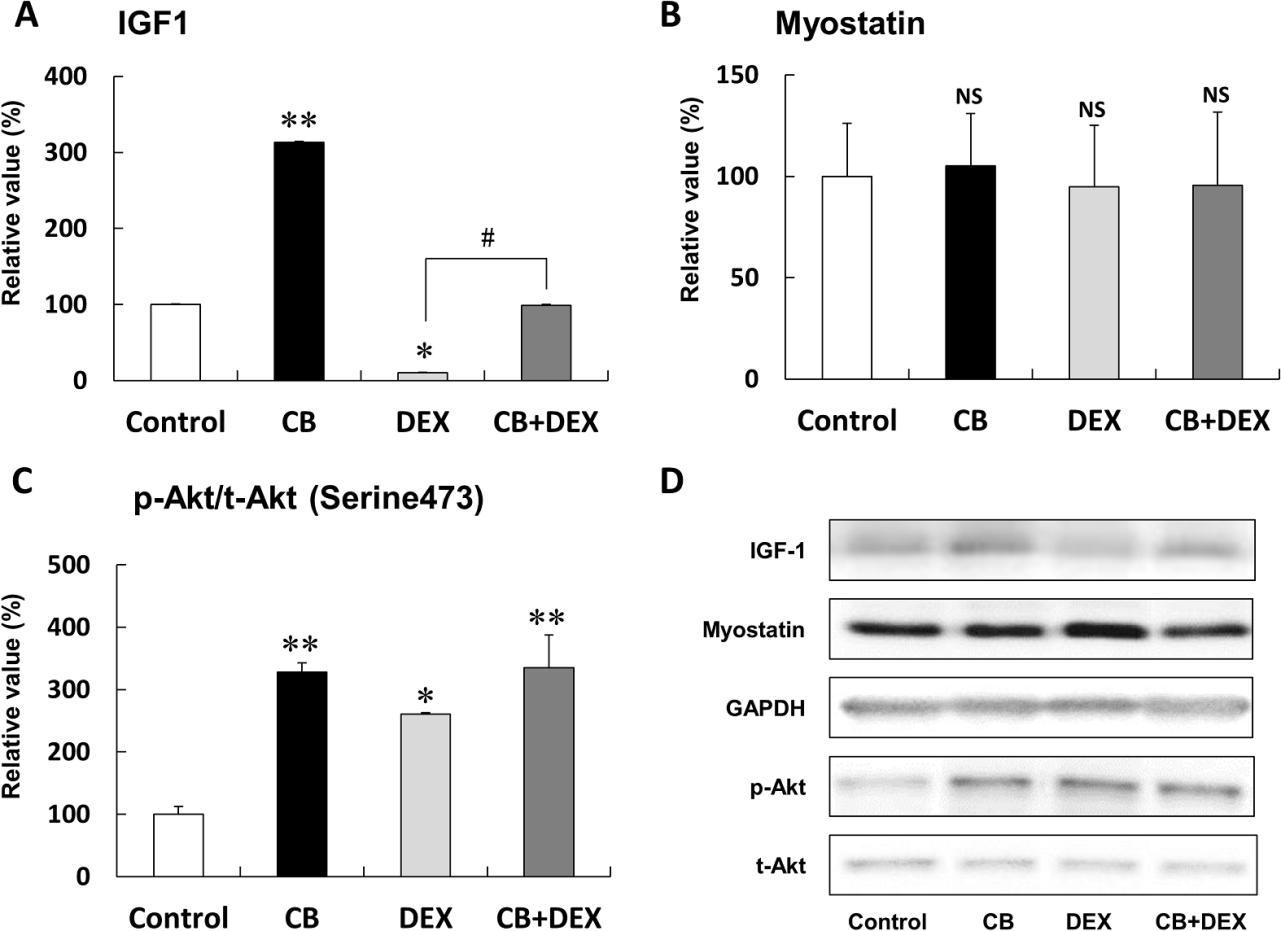
Effects of CB and DEX on IGF1 expression, myostatin expression, and Akt phosphorylation in masseter muscle after treatment with CB and/or DEX for 2 weeks. **(A)** IGF1 expression in masseter muscle of the CB group was greater than that of the Control (**P < 0.01). Conversely, IGF1 expression of the DEX group was smaller than that of the Control (*P < 0.05). The DEX-mediated inhibition of IGF1 was suppressed by co-treatment with CB (CB+DEX vs. DEX, ^#^ *P* < 0.05) **(B)** Expression of myostatin protein was similar in all four groups (*P* = NS vs. Control in each case). **(C)** Phosphorylation of Akt on serine 473 in the CB, DEX, and CB+DEX groups was significantly greater than that in the Control (*P < 0.05, **P < 0.01 vs. Control). **(D)** Representative immunoblotting results for IGF1, myostatin, and phosphorylated Akt, and total Akt. The amount of expression or phosphorylation level in the Control was taken as 100% in each determination. p-Akt, phosphorylated Akt at serine 473; t-Akt, total Akt, GAPDH; glyceraldehyde 3- phosphate dehydrogenase

**Fig 6:**
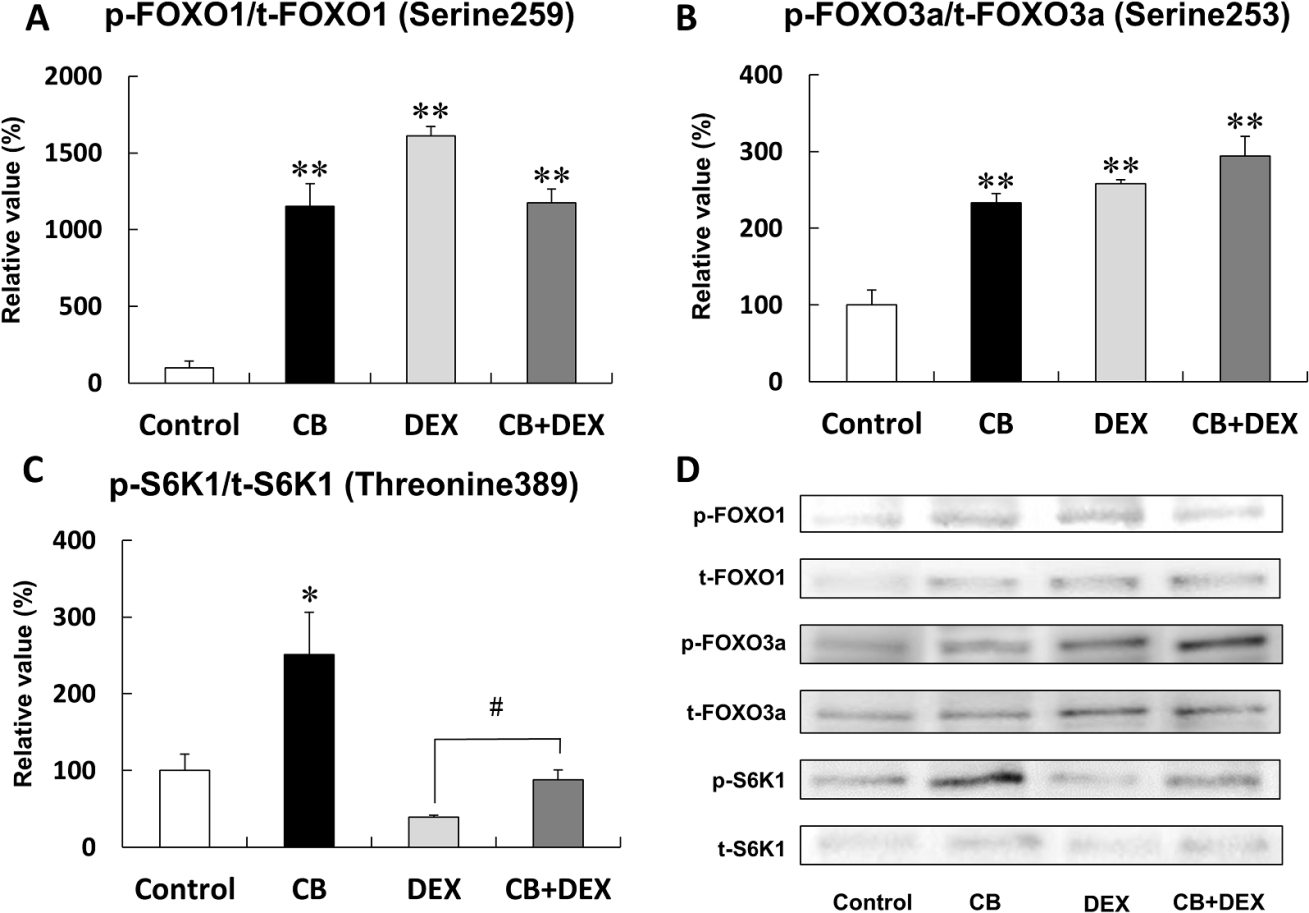
Effects of CB and DEX on phosphorylation of FOXO1, FOXO3a, and S6K1. **(A)** Phosphorylation of FOXO1 at serine 259 in the CB, DEX, and CB+DEX groups was similarly and significantly greater than that in the Control (**P < 0.01 vs. Control in each case). **(B)** Phosphorylation of FOXO3a at serine 253 in CB, DEX, and CB+DEX was similarly and significantly greater than that in the Control (**P < 0.01 vs. Control in each case). **(C)** Phosphorylation of S6K1 on threonine 389 in the CB group was significantly greater than that in the Control (*P < 0.05), while that in the DEX group was significantly lower than that in the Control (*P < 0.05). Importantly, DEX-induced dephosphorylation at this site was significantly suppressed by co-treatment with CB (CB+DEX vs. DEX (^#^ *P* < 0.05)). **(D)** Representative immunoblotting results for phosphorylated and total FOXO1,FOXO3a, and S6K1. The amount of phosphorylation in the Control was taken as 100% in each determination. p-FOXO1, phosphorylated FOXO1 at serine 259; t-FOXO1, total FOXO1; p-FOXO3a, phosphorylated FOXO3a at serine 253; t-FOXO3a, total FOXO3a, p-S6K1, phosphorylated S6K1 at threonine 389; total S6K1, t-S6K1.

**Fig 7:**
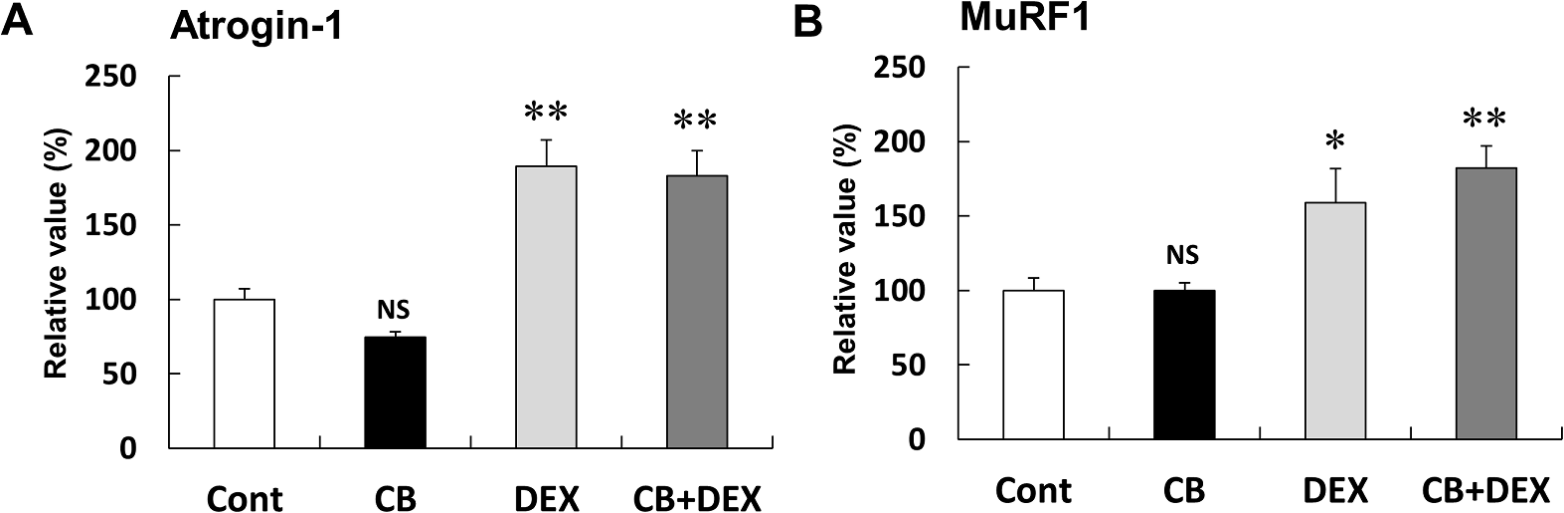
Changes in expression of atrogin-1 and MuRF1 in masseter muscle after treatment with CB and/or DEX for 2 weeks. **(A and B)** Expression levels of atrogin-1 mRNA **(A)** and MuRF1 mRNA **(B)** in the DEX group were greater than those of the Control (*P < 0.05, **P < 0.01 vs. Control in each case). DEX-induced upregulation of atrogin-1 mRNA expression, as well as MuRF1 mRNA expression, was unaffected by co-treatment with CB. The amount of mRNA expression in the Control was taken as 100% in each determination.

**Fig 8:**
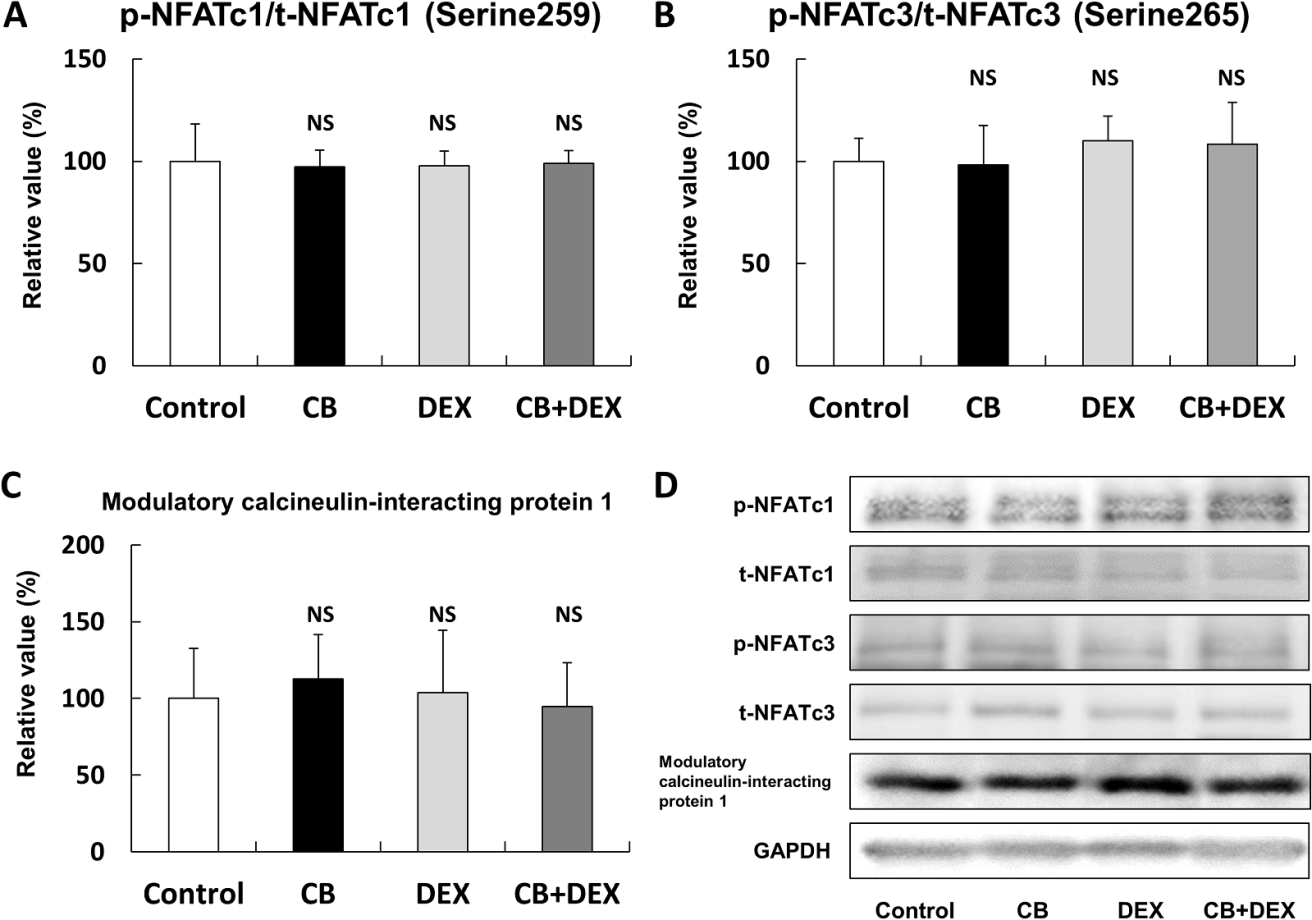
(A-C) Phosphorylation of NFATc1 on serine 259, phosphorylation of NFATc3 on serine 265, and expression of modulatory calcineurin-interacting protein 1 were similar in all four groups (*P* = NS vs. Control in each case). **(D)** Representative immunoblotting results for phosphorylated and total NFATc1 and NFATc3, together with expression of modulatory calcineurin-interacting protein 1. The amount of phosphorylation (NFATc1 and NFATc3) or expression (modulatory calcineurin-interacting protein 1) in the Control was taken as 100% in each determination. p-NFATc1; phosphorylated NFATc1 at serine 259; t-NFATc1; total NFATc1; p-NFATc3, phosphorylated NFATc3 at serine 265, t-NFATc3; total NFATc3, GAPDH; glyceraldehyde 3- phosphate dehydrogenase

All data are expressed as means + S.E.M. After confirming that the variables were normally distributed, we performed inter-group comparisons of body weight (BW), food intake, water intake, CB intake, energy intake (Fig 1), masseter muscle weight, fiber diameter, CSA (Fig 2), MHC (IIa, IId/x, and IIb) protein expression (Fig 3), β_2_-AR, glucocorticoid receptor (Fig 4), IGF1, myostatin, p-Akt (Serine 473)/total Akt (Fig 5), p-FOXO1/total FOXO1 (Serine 259), p-FOXO3a/total FOXO3a (Serine 253), p-S6K1/total S6K1(Serine 473) (Fig 6), atrogin-1, MuRF1 (Fig 7), p-NFATc1/total NFATc1 (serine 259), p-NFATc3/total NFATc3 (Serine 265), modulatory calcineurin-interacting protein (Fig 8) in the four groups (Control, CB, DEX, and CB+DEX) by means of one-way analysis of variance (ANOVA). If a significant difference (*P* < 0.05) among the four groups was found, the parameters were subjected to multiple comparison using Tukey’s post hoc test to confirm a significant difference between two groups. The criterion of significance was taken as *P* < 0.05.

## Results

### Effects of CB and DEX on body weight

We first examined the effects of CB and/or DEX on BW (Fig 1A). CB treatment had no significant effect on BW, in agreement with previous findings (Control (n = 6) vs. CB (n = 9): 371 + 6.4 vs. 379 + 9.4 g, *P* = NS (not significant)) [5,27,28]. Oral administration of CB thus results in significantly greater skeletal muscle mass (see below) without causing a significant difference of total BW compared with the Control group. Since CB is known to promote lipolysis and to decrease fat tissue, we speculate that those changes might compensate at least in part for the increase of the skeletal muscle mass, resulting in no significant difference of total BW between the CB and Control groups [5,9,27–29].

Conversely, BW of both the DEX and CB+DEX groups was significantly lower than that in the Control group (DEX (n = 8): 279 + 4.0 g, CB+DEX (n = 8): 283 + 4.6 g, both *P* < 0.01 vs. Control) [30,31].

### Daily consumption of food and water, oral administration of CB, and intake of energy

We monitored the daily consumption of pellet food (Fig 1B) and water (Fig 1C), and confirmed that the consumed amounts of both pellet food and water were similar among all four groups (food: CB (n = 9): 24 + 1.3 g, DEX (n = 9): 23 + 1.1 g, CB+DEX (n = 9): 27 + 1.4 g, *P* = NS vs. Control (n = 9) in all cases: 27 + 1.0 g; water: CB (n = 9): 32 + 1.5 mL, DEX (n = 9): 31 + 0.5 mL, CB+DEX (n = 9): 32 + 1.5 mL, *P* = NS vs. Control (n = 9) in all cases: 33 + 0.5 mL). This suggests that the taste or odor of CB had no effect on consumption. We also calculated how much CB was administered per baseline BW in the CB and CB+DEX groups (CB (n = 9): 0.0037 + 2.9 x 10^–5^ mg/day/g, CB+DEX (n = 9): 0.0037 + 2.2 x 10^–5^ mg/day/g, *P* = NS) (Fig 1D), as well as the energy intake (Fig 1E) in the four groups (CB (n = 9): 0.32 + 0.002 kcal/day/g, DEX (n = 9): 0.31 + 0.002 kcal/day/g, CB+DEX (n = 9): 0.31 + 0.002 kcal/day/g, *P* = NS vs. Control (n = 9): 0.31 + 0.004 kcal/day/g), and confirmed that they were similar among the groups.

### CB suppressed DEX-induced masseter muscle atrophy

We next examined the effects of CB and DEX on masseter muscle weight. Chronic administration of CB via the drinking water (30 mg/L) for 2 weeks resulted in masseter muscle hypertrophy (Control (n = 9) vs. CB (n = 6): 1281 ± 41 mg vs. 1755 ± 58 mg, *P* < 0.01) (Fig 2A).

Masseter muscle weight in the DEX group was significantly lower than that in the Control (Control (n = 9) vs. DEX (n = 8): 1281 ± 41 mg vs. 1079 + 26 mg, *P* < 0.01) [32]. However, co-treatment with CB suppressed DEX-induced masseter muscle atrophy (DEX (n = 8) vs. CB+DEX (n = 8): 1079 + 26 mg vs. 1470 + 30 mg, *P* < 0.01) (Fig 2A).

Histological analysis showed no abnormal organization of masseter muscle (such as fibrosis) among the four groups (Fig 2B). We also examined masseter muscle hypertrophy in terms of fiber diameter. The fiber diameter in the CB group was significantly greater than that in the Control (Control (n = 104–125 fibers from each of 11 rats) vs. CB (n = 113–257 fibers from each of 11 rats): 50 + 1.3 μm vs. 62 + 0.9 μm, *P* < 0.01) (Fig 2C). Fiber diameter in the DEX group was smaller than that in the Control (Control vs. DEX (n = 163–282 fibers from each of 11 rats): 50 + 1.3 μm vs. 43 + 1.5 μm, *P* < 0.01) (Fig 2C). As in the case of masseter muscle weight, co-treatment with CB suppressed the DEX-mediated reduction of fiber diameter (DEX vs. CB+DEX (n = 114–171 fibers from each of 11 rats): 43 + 1.5 μm vs. 55 + 1.3 μm, *P* < 0.05) (Fig 2C).

We also examined masseter muscle hypertrophy in terms of CSA (Fig 2D). CSA of the CB group was significantly greater than that of the Control group (Control (n = 4) vs. CB (n = 4): 2837 + 220 μm^2^ vs. 3980 + 51 μm^2^, *P* < 0.01), while CSA of the DEX-treated group was significantly lower than that of the Control (Control vs. DEX (n = 4): 2837 + 220 μm^2^ vs. 2063 + 88 μm^2^, *P* < 0.05) (Fig 2D). As in the case of masseter muscle weight and fiber diameter, co-treatment with CB suppressed the DEX-mediated reduction of CSA (DEX vs. CB+DEX (n = 4): 2073 + 88 μm^2^ vs. 2959 + 146 μm^2^, *P* < 0.01) (Fig 2D).

These data collectively indicate that CB significantly suppressed DEX-induced masseter muscle atrophy in rats.

### Effects of CB on DEX-induced muscle atrophy in other muscles

We also examined the weight changes of other skeletal muscles (tibialis anterior and soleus) and cardiac muscle at 2 weeks after CB and/or DEX treatment (Table 1).

**Table 1:** Muscle mass (mg) of rats in Control, CB, DEX or CB+DEX group.

	Tibialis anterior (mg)	Soleus (mg)	Heart (mg)
Control	588 ± 20	141 ± 8	1021 ± 70
CB	741 ± 61 **	165 ± 8 **	1102 ± 37
DEX	452 ± 11 **	114 ± 4 **	952 ± 49
CB+DEX	499 ± 22	123 ± 3 *	1032 ± 55

* p < 0.05,

** p < 0.01, vs. Control, n = 3.

The weight of tibialis anterior and soleus muscles was significantly higher in the CB-treated group than in the Control (n = 3 each, *P* < 0.01). Conversely, the weight of these muscles in the DEX-treated group was significantly lower than in the Control (n = 3 each, *P* < 0.01). In the CB+DEX group, the muscle weights were intermediate between those of the CB and the DEX groups, though the weight of soleus muscle was still significantly lower than that in the Control (*P* < 0.05). Similar changes were observed in cardiac muscle among the four groups, though without statistical significance. These data suggest that CB and DEX at the dose levels used in this study act on other muscles in the same manner as on the masseter muscle. Importantly, these results are consistent with the idea that co-treatment with CB might antagonize DEX-induced muscle atrophy throughout the body.

### CB suppressed DEX-induced fast-to-slow MHC isoform transition

Chronic administration of CB induces slow-to-fast MHC isoform transition [3–5] and chronic administration of DEX induces fast-to-slow MHC isoform transition in skeletal muscle [6]. However, the effect of CB on DEX-induced fast-to-slow MHC isoform transition in the masseter muscle has not been examined. Thus, we analyzed MHC isoform composition at the protein level by means of SDS-PAGE, followed by silver staining and densitometric scanning (Fig 3).

Protein expression of MHC-IIa in masseter muscle of the CB group was significantly lower than that of the Control (Control (n = 8) vs. CB (n = 6): 100 + 10.4% vs. 40 + 3.7%, *P* < 0.01), but no significant change was observed in the DEX group or the CB+DEX group (DEX (n = 8): 98 + 13%, CB+DEX (n = 5): 87 + 9.0%, *P* = NS vs. Control in each case) (Fig 3A).

Protein expression of MHC-IId/x in the CB group (n = 4) was similar to that of the Control (n = 5), but tended to be greater than the Control in the DEX (n = 6) and CB+DEX (n = 8) groups, though without significance (*P* = NS vs. Control in all cases) (Fig 3B).

On the other hand, expression of MHC-IIb in masseter muscle of the CB group was significantly greater than that of the Control (Control (n = 8) vs. CB (n = 8): 100 + 6.5% vs. 141 + 8.5%, *P* < 0.01) (Fig 3C). Conversely, it was lower in the DEX group than in the Control (Control vs. DEX (n = 7): 100 + 6.5% vs. 67 + 8.2%, *P* < 0.01), but the DEX-mediated inhibitory effect on IIb expression was suppressed by co-treatment with CB (DEX vs. CB+DEX (n = 6): 62 + 6.6% vs. 99 + 8.3%, *P* < 0.05) (Fig 3C). Expression of MHC-Neo was barely detectable in densitometric analysis, and expression of MHC-Ia was undetectable, in accordance with our previous findings (Fig 3D) [13].

These data are consistent with the idea that the response to CB in masseter muscle is a sensitive slow-to-fast MHC isoform transition, i.e., a significant increase of MHC-IIb expression with a significant decrease of MHC-IIa expression [5,16], while the response to DEX is intermediate, involving a significant decrease of MHC-IIb without change of MHC-IIa expression, as has been reported in the case of diaphragm [6,33]. This in turn may suggest that the effect of CB on MHC isoform transition is predominant over that of DEX, implying that CB can significantly and effectively antagonize DEX-induced MHC isoform transition toward slower isoforms.

### Effect of CB on DEX-induced atrophy was not mediated by changes of β_2_-AR or glucocorticoid receptor expression

It was reported that β-agonists may alter the expression of glucocorticoid receptor, while glucocorticoids may alter the expression of β-AR [34]. We thus examined the protein expression of β_2_-AR **(** Fig 4A) and glucocorticoid receptor (Fig 4B) by means of western blotting. Expression of β_2_-AR protein was inhibited by CB (Control (n = 3) vs. CB (n = 3): 100 + 20% vs. 31 + 9.9%, *P* < 0.05) and by DEX (Control (n = 3) vs. DEX (n = 3): 100 + 20% vs. 36 + 8.2%, *P* < 0.01). Importantly, co-treatment with CB did not influence the DEX-induced inhibition of β_2_-AR expression (DEX vs. CB+DEX: 36 + 8.2% vs. 20 + 4.0%, *P* = NS). These data suggest that chronic administration of CB or DEX might independently down-regulate β_2_-AR. CB (n = 4), DEX (n = 4) and CB+DEX (n = 4) did not alter expression of glucocorticoid receptor protein (*P* = NS vs. Control in each case).

These data may mean that the co-treatment effect of CB on DEX-induced atrophy is not mediated by change of β_2_-AR or glucocorticoid receptor.

### CB suppressed DEX-induced decrease of IGF1 protein

We examined the effects of CB and DEX on protein expression of IGF1 and myostatin, which are positive and negative regulators of muscle growth by means of western blotting.

IGF1 protein expression in masseter muscle of the CB group was greater than that of the Control group (Control (n = 3) vs. CB (n = 3): 100 + 3.0% vs. 314 + 39%, *P* < 0.01) (Fig 5A and 5D). Conversely, IGF1 protein expression in masseter muscle of the DEX group was lower than that of the Control group (Control vs. DEX (n = 3): 100 + 3.0% vs. 10 + 1.6%, *P* < 0.01). However, the DEX-mediated inhibitory effect on IGF1 expression was suppressed by co-treatment with CB (DEX vs. CB+DEX (n = 3): 10 + 1.6% vs. 99 + 2.3%, *P* < 0.05 vs. DEX). On the other hand, myostatin expression was similar in all four groups (*P* = NS, n = 5 in each case) (Fig 5B and 5D).

These data indicated that augmented IGF1 protein expression might be associated with CB-induced masseter muscle hypertrophy. Conversely, inhibition of IGF1 protein expression might be associated with DEX-induced masseter muscle atrophy. Importantly, co-treatment with CB appears to antagonize the DEX-induced inhibitory effect on IGF1 expression, and this may account in part for the inhibition of DEX-induced muscle atrophy in the CB+DEX group.

### Effects of CB and DEX on Akt phosphorylation on serine 473

We next examined the phosphorylation of Akt on serine 473, because activation of IGF1 receptor signaling is known to induce phosphorylation at this site (Fig 5C and 5D) [35]. Phosphorylation of Akt on serine 473 in the CB group was significantly greater than that in the Control, in accordance with our previous work (CB (n = 3) vs. Control (n = 3): 328 + 15% vs. 100 + 13%, *P* < 0.01) [16], and that in the DEX group was also greater than that in the Control (DEX (n = 3) vs. Control (n = 3): 260 + 15% vs. 100 + 13%, *P* < 0.05), though the phosphorylation induced by CB and DEX (CB+DEX (n = 3) in Fig 5C) was not additive, possibly because Akt phosphorylation on serine 473 might be saturated in response to CB.

These data are consistent with the idea that both CB and DEX similarly induce phosphorylation of Akt on serine 473 in masseter muscle.

### Akt/FOXO pathway was similarly activated by CB and DEX

We next examined the phosphorylation at serine 259 of FOXO1 (Fig 6A) and at serine 253 of FOXO3a (Fig 6B), which are downstream of Akt (Akt/FOXO pathway) and might regulate the muscle atrophy-related genes, such as atrogin-1 and MuRF1 [13,36].

Phosphorylation of FOXO1 on serine 259 was greater in masseter muscle of both the CB group (CB (n = 3) vs. Control (n = 3): 1152 + 149% vs. 100 + 44%, *P* < 0.01) and the DEX group (DEX (n = 3) vs. Control (n = 3): 1610 + 61% vs. 100 + 44%, *P* < 0.01), compared with the Control, though the effects of CB and DEX were not additive, as in the case of Akt phosphorylation on serine 473 (Fig 6A and 6D).

Phosphorylation of FOXO3a on serine 253 was also greater in masseter muscle of both the CB group (CB (n = 3) vs. Control (n = 3): 233 + 12% vs. 100 + 19%, *P* < 0.01) and the DEX group (DEX (n = 3) vs. Control (n = 3): 258 + 5% vs. 100 + 19%, *P* < 0.01), compared with the Control (Fig 6B and 6D).

These data are consistent with the idea that both CB and DEX similarly and significantly activate the Akt/FOXO pathway in masseter muscle.

### Effects of CB and DEX on the Akt/mTOR pathway

In order to investigate molecular signaling involved in the antagonistic effect of CB on DEX-induced masseter muscle atrophy, we examined activation of the Akt/mTOR pathway by evaluating phosphorylation of S6K1, a major target of the Akt/mTOR pathway and a well-documented hypertrophic signal, after treatment with CB and/or DEX for 2 weeks [13,16].

Phosphorylation of S6K1 on threonine 389 in the masseter muscle of CB-treated rats was significantly greater than that of the Control (CB (n = 3) vs. Control (n = 3): 251 + 55% vs. 100 + 21%, *P* < 0.01). Conversely, phosphorylation at this site was significantly smaller in the DEX group than in the Control (DEX (n = 3) vs. Control (n = 3): 39 + 3% vs. 100 + 21%, *P* < 0.05), and the DEX-mediated inhibitory effect was suppressed by co-treatment with CB (DEX (n = 3) vs. CB+DEX (n = 3): 39 + 3% vs. 88 + 13%, *P* < 0.05) (Fig 6C and 6D).

These data appear to indicate that co-treatment of CB might attenuate DEX-induced masseter muscle atrophy through activation of the Akt/mTOR pathway.

### CB had no effect on DEX-induced upregulation of atrogin-1 and MuRF1 mRNAs

In order to examine the effects of DEX on the expression of atrogin-1 and MuRF1, which are downstream molecules of the Akt/FOXO pathway and also mediate muscle atrophy, we examined mRNA expressions of these molecules in masseter muscle **(** Fig 7A and 7B) [37].

Expression of atrogin-1 mRNA, as well as MuRF1 mRNA, was unaffected by CB treatment (atrogin-1: Control (n = 10) vs. CB (n = 8): 100 + 7.1% vs. 75 + 3.8%, *P* = NS); MuRF1: Control (n = 10) vs. CB (n = 8): 100 + 8.5% vs. 100 + 5.6%, *P* = NS). However, both mRNAs in the DEX-treated group were significantly greater than those in the Control, and CB had no influence on the effect of DEX (atrogin-1: DEX (n = 8) vs. CB+DEX (n = 8): 189 + 17.7% vs. 183 + 16.9%, *P* = NS; MuRF1: DEX (n = 8) vs. CB+DEX (n = 8): 159 + 22.7% vs. 182 + 14.7%, *P* = NS).

These data, together with the data shown in Fig 6A and 6B, suggest that Akt/FOXO activation induced by DEX may contribute to the positive regulatory effects on expression of atrogin-1 mRNA and MuRF1 mRNA through a pathway that is independent of CB-mediated Akt/FOXO activation.

### Calcineurin-NFAT signaling was not altered by CB or DEX treatment

Calcineurin is a calcium/calmodulin-regulated protein phosphatase that acts on the transcription factors of the NFAT family, causing them to be translocated to the nucleus, where they induce transcriptional activation [38–40]. We and another group have shown that calcineurin-NFAT signaling has a role in preservation of muscle mass, as well as MHC fiber type switching [41,42].

We thus examined the effects of CB and DEX on calcineurin-NFAT signaling and found that the phosphorylation of NFATc1 on serine 259 (Fig 8A and 8D) and NFATc3 on serine 265 (Fig 8B and 8D) were similar in all four groups (n = 5 each, *P* = NS).

Modulatory calcineurin-interacting protein 1, which is highly expressed in skeletal muscle, is induced by calcineurin and it inhibits calcineurin activity, thereby establishing a negative feedback loop [13]. We thus examined the effects of CB and DEX on expression of modulatory calcineurin-interacting protein 1 in masseter muscle and found that the expression levels were similar in the four groups (n = 4 each, *P* = NS) (Fig 8C and 8D).

These data are consistent with the idea that CB and DEX do not influence activation of calcineurin-NFAT signaling in masseter muscle.

## Discussion

Glucocorticoids such as DEX are widely used in clinical medicine, but may cause the serious adverse effect of glucocorticoid-induced myopathy. Recently, we demonstrated that CB induces slow-to-fast MHC transition together with masseter muscle hypertrophy through direct muscle β_2_-AR stimulation, whereas the increase of daily duty time was mediated through the central nervous system [5]. Therefore, we speculated that CB might antagonize DEX-induced muscle atrophy, and might be available as a new pharmacotherapy for the treatment of steroid-induced myopathy. In order to test this idea, we first confirmed the CB-mediated protective effect against DEX-induced muscle atrophy, as well as the inhibitory effect of CB on DEX-induced MHC isoform transition toward slower isoforms in rats. The protective effect appears to be due to an interaction between CB and DEX, but the mechanism involved was unclear

Information regarding the action of β_2_-agonist on glucocorticoid-stimulated anti-inflammatory effects is conflicting, at least in part. Reciprocal inhibition of glucocorticoid response element (GRE) by β_2_-AR agonist, and of cyclic AMP (cAMP) response elements (CRE) by glucocorticoid has been reported [43]. On the other hand, β_2_-AR agonist can enhance the anti-inflammatory effect of GRE [44,45]. However, less attention has been paid to the interaction between β_2_-AR agonist and glucocorticoid in skeletal muscle, even though both classes of compounds are frequently prescribed for treatment of asthma. Importantly, they are both very effective on skeletal muscle, but have opposite effects: β_2_-AR promotes muscle growth and hypertrophy [5,16], whereas glucocorticoids induce muscle atrophy and loss of contractile strength [2]. Therefore, we investigated the mechanisms involved in the protective effects of CB against DEX-induced muscle atrophy.

**Fig 9:**
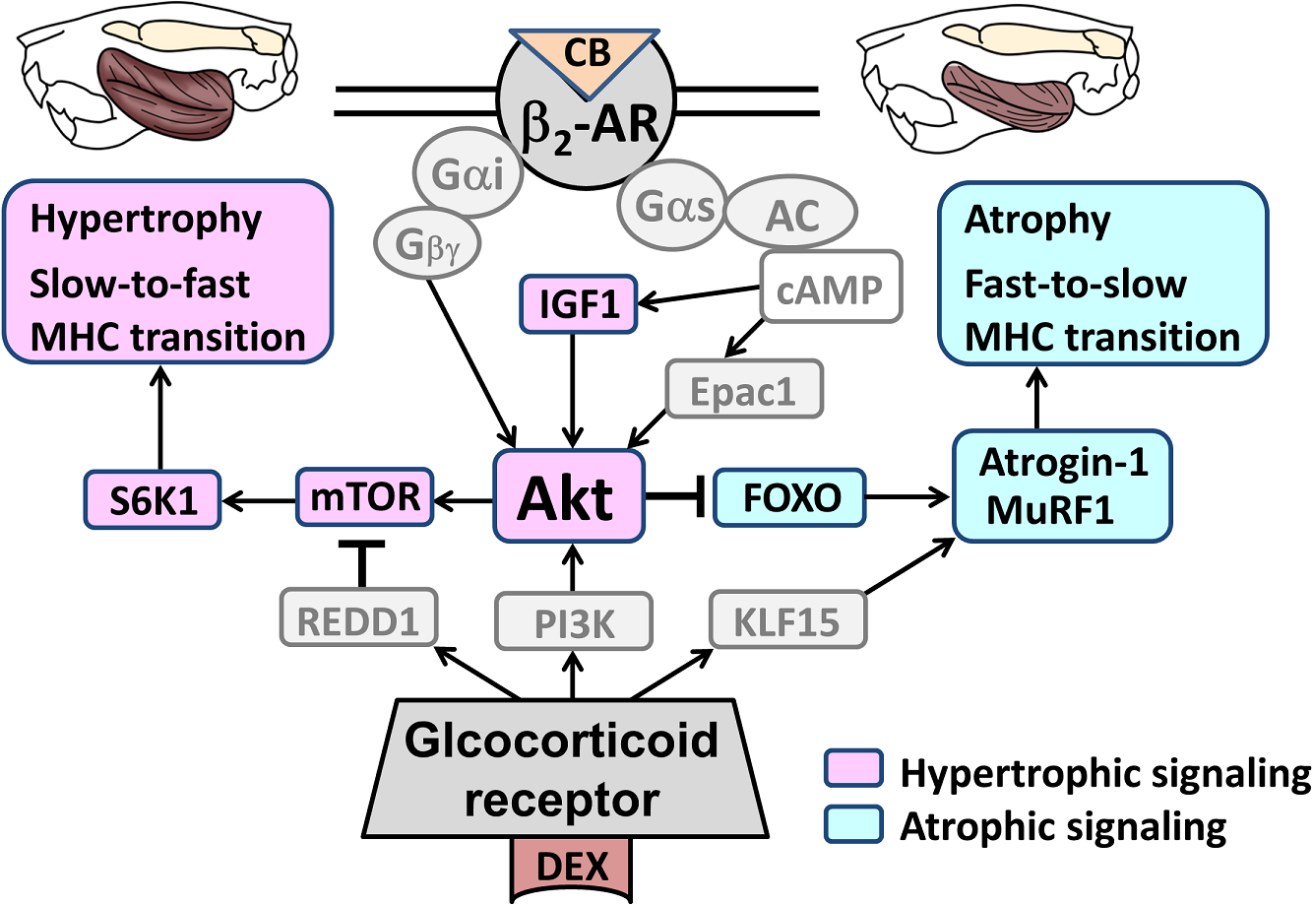
Schematic illustration of the proposed pathways involved in the protective effects of CB against DEX-induced muscle atrophy. CB as well as DEX induces phosphorylation of Akt on serine 476. Activation of the Akt/mTOR pathway was increased by CB, but decreased by DEX. Activation of the Akt/FOXO pathway was similarly increased by CB and DEX, indicating that another pathway that is independent of Akt/FOXO pathway may account for the augmented expression of atrogin-1 and MuRF1 in DEX-treated group. Solid black lines represent findings in this study and solid grey lines represent findings reported previously [5,13,16,48]. IGF1; insulin growth factor 1, REDD1; regulated in development and DNA damage responses 1, PI3K; phosphoinositide 3-kinase, KLF15; Kruppe-like factor 15

It was recently reported by us that Akt/mTOR pathway is involved in masseter muscle hypertrophy, and extracellular signaling regulated kinase 1/2 phosphorylation exerts an opposing effect on mechanical-overload-induced masseter muscle hypertrophy [13]. We also examined the effect of disruption of Epac1 (exchange protein directly activated by cAMP), which was recently identified as a new PKA-independent cAMP sensor and a major skeletal muscle isoform [14]; our results indicated that CB-mediated masseter muscle hypertrophy might develop as a result of activation of cAMP/Epac1/Akt signaling, rather than cAMP/PKA signaling [46], because the cAMP/PKA signaling in masseter muscle was intact in both WT and Epac1KO [14,16]. We thus examined the phosphorylation of Akt on serine 473 and found that phosphorylation of Akt on serine 473 in the CB group was significantly greater than that in the Control in both CB-treated and DEX-treated groups. Thus, Akt phosphorylation in the CB group might be mediated via β_2_-AR/cAMP/Epac1/Akt signaling, in agreement with our previous findings in Epac1KO (Fig 9) [16]. As for Akt phosphorylation in the DEX-treated group, we anticipated that this might be mediated via activation of the phosphoinositide 3-kinase/Akt pathway, which was reported to be a neuroprotective signaling pathway ameliorating DEX-induced hypoxic-ischemia brain injury in newborn rats [47] (Fig 9).

Next, we examined and confirmed the phosphorylation on serine 259 of FOXO1 and on serine 253 of FOXO3a, which are downstream of Akt (Akt/FOXO pathway) and might regulate muscle atrophy-related genes, such as atrogin-1 and MuRF1, in the CB- and/or DEX-treated groups [13,36]. Indeed, expression levels of atrogin-1 and MuRF1 were each significantly increased in both the DEX- and CB+DEX-treated groups, compared with the Control group, suggesting that upregulation of atrogin-1 and MuRF1 in response to DEX might be mediated through an Akt/FOXO-independent pathway, such as Kruppe-like factor 15 (KLF15), a recently discovered transcription factor that promotes skeletal muscle atrophy via transcriptional regulation of atrogin-1 and MuRF1 in DEX-treated rats (Fig 9 **)** [48].

We thus hypothesized that the mechanisms through which CB antagonizes DEX-induced muscle atrophy might be associated with activation of the Akt/mTOR pathway, which is another major signaling pathway for muscle hypertrophy [16]. Importantly, co-treatment with CB significantly attenuated DEX-mediated S6K1 dephosphorylation. It was reported that translational repressor protein REDD1 (regulated in development and DNA damage responses 1) is induced by various stresses, including glucocorticoid receptor activation, and induces muscle atrophy via inhibition of the Akt/mTOR pathway [48]. We speculate that REDD1 might be associated with the attenuated Akt/mTOR signaling in the DEX-treated group in this study (Table 1).

It has also been reported that IGF1 induces skeletal muscle hypertrophy and myostatin induces muscle atrophy [36,49]. We found that expression of IGF1 protein was significantly attenuated by DEX, but significantly augmented by CB. However, expression of myostatin showed no significant difference among the four groups. These data indicate that co-treatment with CB might antagonize the DEX-induced inhibitory effect on IGF1 expression, and this effect of CB might also be associated with its protective effect against DEX-induced muscle atrophy, in addition to activation of the Akt/mTOR pathway.

Taken together, our experimental results indicate that CB antagonizes DEX-induced muscle atrophy with fast-to-slow MHC isoform transition by blocking the DEX-induced inhibitory effect on Akt/mTOR activity and IGF-1 expression (Fig 9). We believe these findings could provide the basis for a new pharmacological approach to the treatment of glucocorticoid-induced muscle atrophy.
